# Crystal structure and Hirshfeld surface analysis of (*E*)-*N*-(4-propyl­oxybenzyl­idene)benzo[*d*]thia­zol-2-amine

**DOI:** 10.1107/S2056989020012128

**Published:** 2020-09-04

**Authors:** Ropak A. Sheakh Mohamad, Wali M. Hamad, Hashim J. Aziz, Necmi Dege

**Affiliations:** a Salahaddin University, College of Science, Department of Chemistry, Erbil, Iraq; b Koya University, Faculty of Science and Health, Department of Chemistry, Koya, Iraq; c Salahaddin University, College of Education, Department of Chemistry, Erbil, Iraq; d Ondokuz Mayıs University, Faculty of Arts and Sciences, Department of Physics, 55139, Samsun, Turkey

**Keywords:** crystal structure, heterocyclic compound, 2-amino­benzo­thia­zole, Schiff base, Hirshfeld surface

## Abstract

The title compound, C_17_H_16_N_2_OS, was synthesized by a condensation reaction between 2-amino benzo­thia­zole and 4-*N*-propoxybenzaldehyde. The benzo[*d*]thia­zole ring system is nearly planar (r.m.s. deviation 0.0088 Å) and makes a dihedral angle of 3.804 (12)° with the phenyl ring. The configuration about the C=N double bond is *E*. In the crystal structure, pairs of C—H⋯N hydrogen bonds and C—H⋯π inter­actions link the mol­ecules into inversion dimers with an *R* ^2^_2_(16) ring motif. These dimers are additionally linked by weak π–π stacking inter­actions between the phenyl rings, leading to a layered arrangement parallel to (010). Hirshfeld surface analysis of the crystal structure indicates that the most important contributions for the packing arrangement are from H⋯H (47.9%) and C⋯H/H⋯C (25.6%) inter­actions.

## Abstract 2

In the crystal, the mol­ecules are linked by C—H⋯N and weak C—H⋯π hydrogen bonds and very weak π–π stacking inter­actions. Two-dimensional fingerprint plots show that the largest contributions to the crystal stability come from H⋯H and C⋯H/H⋯C inter­actions.

## Chemical context   

Benzo­thia­zole is one of the most important heterocyclic compounds, comprising of a sulfur and a nitro­gen atom that constitute the core structure of thia­zole. Benzo­thia­zole is a weak base, and is widely found in bioorganic and medicinal chemistry with application in drug discovery as a pharmacologically and biologically active compound (Quin & Tyrell, 2010 ▸). Benzo­thia­zole and its derivatives show numerous biological activities such as anti­microbial, anti­cancer, anthelmintic or anti-diabetic. They have also found application in industry as anti­oxidants and vulcanization accelerators (Achaiah *et al.*, 2016 ▸).
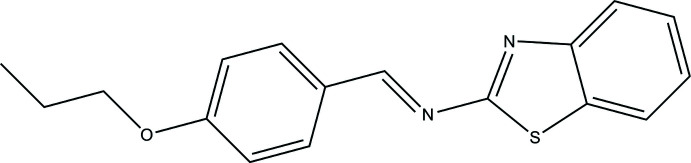


Schiff bases (Schiff, 1864 ▸) are nitro­gen analogues of aldehydes or ketones in which the corresponding functional group has been replaced by an imine or azomethine group. They can be synthesized from the reaction of primary amines with an aldehyde or a ketone under particular conditions. Schiff bases are some of the most widely used organic compounds, utilized, for example, as catalysts, pigments and dyes, inter­mediates in organic synthesis, or as polymer stabilizers. Moreover, Schiff bases exhibit a broad range of biological activities such as anti­viral, anti­bacterial, anti-inflammatory, anti­malarial, anti­fungal, anti-proliferative and anti­pyretic properties (Bhoi *et al.*, 2015 ▸).

In the context given above, we report here the synthesis, mol­ecular and crystal structure of the Schiff base C_17_H_16_N_2_OS, comprising a benzo­thia­zole moiety.

## Structural commentary   

**Figure 1:**
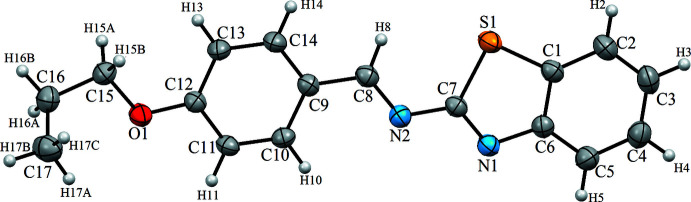
The mol­ecular structure of the title compound, with atom labelling. Displacement ellipsoids are drawn at the 40% probability level.

The asymmetric unit of the title compound is comprised of one mol­ecule (Fig. 1 ▸), which exhibits an *E* configuration for the imine functionality. The benzo[*d*]thia­zole ring system is nearly planar [r.m.s. deviation 0.0088 Å, with the largest deviation being 0.0127 (18) Å for atom C4]. The benzo[*d*]thia­zole ring system and the phenyl ring (C9–C14) are slightly twisted with respect to each other, making a dihedral angle of 3.804 (12)°. In the thia­zole ring, the C6—N1 [1.379 (3) Å] and C7—N1 [1.288 (3) Å] distances indicate substantial electronic delocalization. The C8=N2 double bond has a length of 1.272 (3) Å, and thus is slightly longer than comparable bonds found in other Schiff base structures (Sen *et al.*, 2018 ▸; Kansiz *et al.*, 2018 ▸), which are in the range of 1.262 (3)–1.270 (3) Å. The methyl group of the propyl chain is moved out by 59.2 (3)° from the mean plane of the rest of the mol­ecule.

## Supra­molecular features   

**Figure 2:**
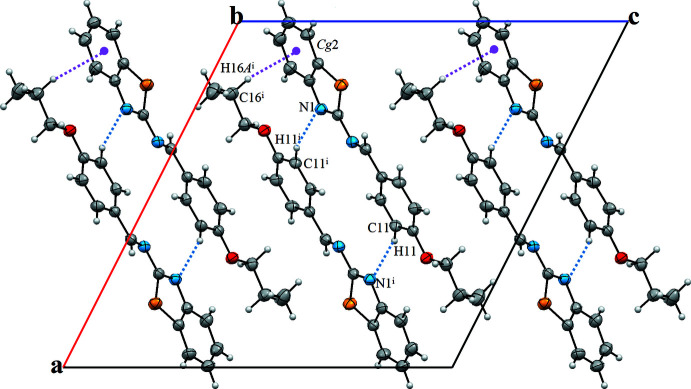
A view of the crystal packing of the title compound. Inter­molecular inter­actions are displayed by dotted lines. The symmetry code refers to Table 1 ▸.

In the crystal structure, mol­ecules are linked by C—H⋯π hydrogen bonds (Table 1 ▸) between one of the methyl­ene C atoms of the propyl group (C16—H16*A*) and the centroid of the C1–C6 phenyl ring (*Cg*2) of an adjacent mol­ecule (Fig. 2 ▸). Pairs of additional C—H⋯N hydrogen bonds form inversion dimers with an 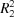
(16) ring motif (Fig. 2 ▸). The dimers are additionally linked by weak π–π inter­actions, with a centroid-to-centroid distance of 4.695 (2) Å between *Cg*3 and *Cg*3^i^ [Symmetry code: (i): −*x* + 1, −*y* + 2, −*z*) where *Cg*3 is the centroid of the C9–C14 phenyl ring. The resulting supra­molecular network is layered and expands parallel to (010).

## Hirshfeld surface analysis   

**Figure 3:**
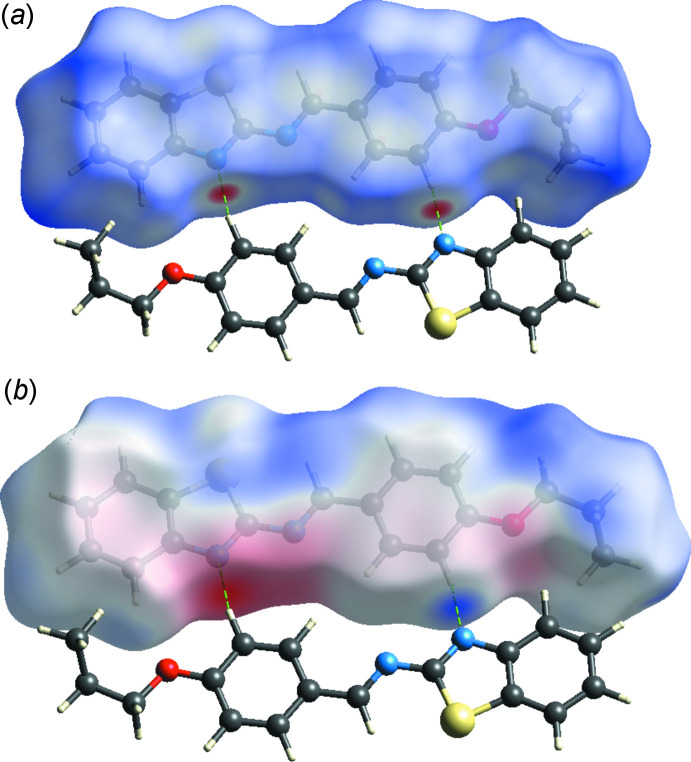
The Hirshfeld surface of the title compound mapped over (*a*) *d*_norm_ and (*b*) electrostatic potential, showing the C—H⋯N hydrogen bond.

**Figure 4:**
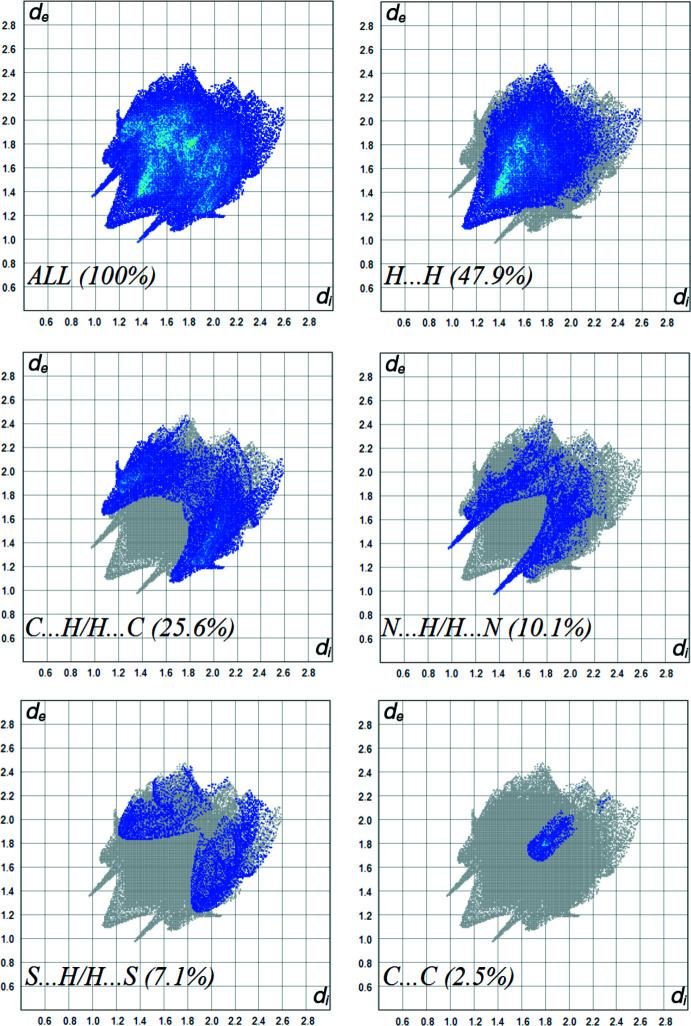
Two-dimensional fingerprint plots, showing the relative contribution of the atom-pair inter­actions to the Hirshfeld surface.

Hirshfeld surface analysis together with two-dimensional fingerprint plots are a powerful tool for the visualization and inter­pretation of inter­molecular contacts in mol­ecular crystals (Spackman & Jayatilaka, 2009 ▸). The corresponding surfaces and fingerprint plots were obtained using *CrystalExplorer* (Turner *et al.*, 2017 ▸). The *d*_norm_ and mol­ecular electrostatic potential maps for the title compound are shown in Fig. 3 ▸ *a* and 3*b*, respectively, with the prominent hydrogen-bonding inter­actions shown as red spots. The red spots identified in Fig. 3 ▸ *a* correspond to the H⋯N contacts resulting from hydrogen bond C—H⋯N (Table 1 ▸). The most important contribution to the Hirshfeld surface comes from H⋯H contacts with 47.9%. C⋯H and N⋯H inter­actions follow with 25.6% and 10.1% contributions, respectively (Fig. 4 ▸). Other minor contributors are S⋯H/H⋯S (7.1%), C⋯C (2.5%), O⋯H/H⋯O (2.1%), C⋯N/N⋯C (1.8%), C⋯S/S⋯C (1.1%) and C⋯O/O⋯C (0.8%).

## Database survey   

A search of the Cambridge Structural Database (CSD, version 5.41, update of November 2019; Groom *et al.*, 2016 ▸) for an (*E*)-*N*-benzyl­idenebenzo[*d*]thia­zol-2-amine skeleton gave 20 hits. Of these 20, the most similar to the title compound are 2-[(6-meth­oxy-1,3-benzo­thia­zol-2-yl)carbonoimido­yl]phenol (SUFFEG; Hijji *et al.*, 2015 ▸), (*E*)-2-[(6-eth­oxy­benzo­thia­zol-2-yl)imino­meth­yl]-6-meth­oxy­phenol (VOQKAO; Kong, 2009 ▸) and 2-[(1,3-benzo­thia­zol-2-yl­imino)­meth­yl]phenol (VOQXOP01; Asiri *et al.*, 2010 ▸). All these compounds have an *E* configuration about the C=N imine bond, and have similar bond lengths and angles as mentioned above for the title compound.

## Synthesis and crystallization   

2-Amino benzo­thia­zole (0.3 g, 2 mmol) was dissolved in 10 ml of 1-propanol in a 50 ml borosilicate glass beaker. 4-*N*-Propoxybenzaldehyde (0.328 g, 2 mmol) was then added dropwise into the mixture under stirring, in the presence of a catalytic amount of glacial acetic acid. The reaction mixture was then placed inside an unmodified household microwave oven and was irradiated for 32 min (eight pulses each of 4 min) at 540 W power, with short inter­ruptions of one minute. The progress of the reaction was monitored by thin-layer chromatography using ethyl acetate and *n*-hexane (3:7 *v*:*v*) as eluent (*R*_f_ = 0.69). The formed precipitate was filtered off, washed with 1-propanol, and dried. The resulting solid was further purified by recrystallization from *n*-hexane to give the pure imine as a crystalline solid (yield: 72.4%, m.p. 357–358 K).

## Refinement   

Crystal data, data collection and structure refinement details are summarized in Table 2 ▸. C-bound H atoms were placed in idealized positions and refined using a riding model with C—H = 0.93–0.97 Å with *U*_iso_(H) = 1.5*U*_eq_(C-meth­yl) and 1.2*U*_eq_(C) for other C–bound H atoms.

## Supplementary Material

Crystal structure: contains datablock(s) I. DOI: 10.1107/S2056989020012128/wm5582sup1.cif

Structure factors: contains datablock(s) I. DOI: 10.1107/S2056989020012128/wm5582Isup2.hkl

Supporting information file. DOI: 10.1107/S2056989020012128/wm5582Isup3.cml

CCDC reference: 1979807

Additional supporting information:  crystallographic information; 3D view; checkCIF report

## Figures and Tables

**Table 1:** Hydrogen-bond geometry (Å, °) *Cg*2 is the centroid of the C1–C6 phenyl ring.

*D*—H⋯*A*	*D*—H	H⋯*A*	*D*⋯*A*	*D*—H⋯*A*
C11—H11⋯N1^i^	0.93	2.49	3.362 (3)	157
C16—H16*A*⋯*Cg*2^ii^	0.97	2.91 (2)	3.765 (3)	147

Symmetry codes: (i) 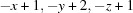; (ii) 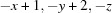.

**Table 2:** Experimental details

Crystal data	
Chemical formula	C_17_H_16_N_2_OS
*M*_r_	296.38
Crystal system, space group	Monoclinic, *P*2_1_/*c*
Temperature (K)	296
*a*, *b*, *c* (Å)	17.251 (1), 5.6849 (3), 17.3101 (11)
β (°)	116.958 (4)
*V* (Å^3^)	1513.14 (16)
*Z*	4
Radiation type	Mo *K*α
μ (mm^−1^)	0.21
Crystal size (mm)	0.67 × 0.34 × 0.04

Data collection	
Diffractometer	Stoe IPDS 2
Absorption correction	Integration (*X-RED32*; Stoe & Cie, 2002 ▸)
*T*_min_, *T*_max_	0.896, 0.983
No. of measured, independent and observed [*I* > 2σ(*I*)] reflections	10315, 2962, 1950
*R*_int_	0.048
(sin θ/λ)_max_ (Å^−1^)	0.617

Refinement	
*R*[*F* ^2^ > 2σ(*F* ^2^)], *wR*(*F* ^2^), *S*	0.045, 0.099, 0.98
No. of reflections	2962
No. of parameters	191
H-atom treatment	H-atom parameters constrained
Δρ_max_, Δρ_min_ (e Å^−3^)	0.14, −0.13

Computer programs: *X-AREA* and *X-RED32* (Stoe & Cie, 2002 ▸), *SHELXT2017/1* (Sheldrick, 2015*a* ▸), *SHELXL2017/1* (Sheldrick, 2015*b* ▸), *PLATON* (Spek, 2020 ▸) and *WinGX* (Farrugia, 2012 ▸).

## supplementary crystallographic information

### Crystal data

**Table:**

C_17_H_16_N_2_OS	*F*(000) = 624
*M**_r_* = 296.38	*D*_x_ = 1.301 Mg m^−^^3^
Monoclinic, *P*2_1_/*c*	Mo *K*α radiation, λ = 0.71073 Å
*a* = 17.251 (1) Å	Cell parameters from 9824 reflections
*b* = 5.6849 (3) Å	θ = 2.4–28.1°
*c* = 17.3101 (11) Å	µ = 0.21 mm^−^^1^
β = 116.958 (4)°	*T* = 296 K
*V* = 1513.14 (16) Å^3^	Plate, yellow
*Z* = 4	0.67 × 0.34 × 0.04 mm

### Data collection

**Table:**

Stoe IPDS 2 diffractometer	2962 independent reflections
Radiation source: sealed X-ray tube, 12 x 0.4 mm long-fine focus	1950 reflections with *I* > 2σ(*I*)
Detector resolution: 6.67 pixels mm^-1^	*R*_int_ = 0.048
rotation method scans	θ_max_ = 26.0°, θ_min_ = 2.4°
Absorption correction: integration (X-RED32; Stoe & Cie, 2002)	*h* = −21→21
*T*_min_ = 0.896, *T*_max_ = 0.983	*k* = −7→7
10315 measured reflections	*l* = −21→19

### Refinement

**Table:**

Refinement on *F*^2^	0 restraints
Least-squares matrix: full	Hydrogen site location: inferred from neighbouring sites
*R*[*F*^2^ > 2σ(*F*^2^)] = 0.045	H-atom parameters constrained
*wR*(*F*^2^) = 0.099	*w* = 1/[σ^2^(*F*_o_^2^) + (0.0451*P*)^2^] where *P* = (*F*_o_^2^ + 2*F*_c_^2^)/3
*S* = 0.98	(Δ/σ)_max_ < 0.001
2962 reflections	Δρ_max_ = 0.14 e Å^−^^3^
191 parameters	Δρ_min_ = −0.13 e Å^−^^3^

### Special details

**Table:**

Geometry. All esds (except the esd in the dihedral angle between two l.s. planes) are estimated using the full covariance matrix. The cell esds are taken into account individually in the estimation of esds in distances, angles and torsion angles; correlations between esds in cell parameters are only used when they are defined by crystal symmetry. An approximate (isotropic) treatment of cell esds is used for estimating esds involving l.s. planes.

### Fractional atomic coordinates and isotropic or equivalent isotropic displacement parameters (Å^2^)

**Table:**

	*x*	*y*	*z*	*U*_iso_*/*U*_eq_	
S1	0.18402 (4)	0.57314 (11)	0.34622 (4)	0.0693 (2)	
O1	0.68408 (9)	0.4674 (3)	0.79162 (9)	0.0670 (4)	
N2	0.34892 (11)	0.7538 (3)	0.44924 (11)	0.0594 (4)	
N1	0.25411 (11)	0.9575 (3)	0.32624 (11)	0.0605 (4)	
C7	0.27040 (13)	0.7771 (4)	0.37623 (13)	0.0548 (5)	
C9	0.44990 (12)	0.5343 (4)	0.57024 (12)	0.0536 (5)	
C12	0.60529 (13)	0.4773 (4)	0.72070 (12)	0.0543 (5)	
C8	0.36920 (13)	0.5628 (4)	0.49215 (13)	0.0594 (5)	
H8	0.330649	0.436935	0.472533	0.071*	
C6	0.17124 (13)	0.9462 (4)	0.25862 (13)	0.0574 (5)	
C1	0.12228 (13)	0.7506 (4)	0.25832 (13)	0.0591 (5)	
C10	0.51498 (13)	0.7056 (4)	0.59654 (12)	0.0570 (5)	
H10	0.506190	0.841213	0.563558	0.068*	
C11	0.59124 (13)	0.6761 (4)	0.67003 (13)	0.0581 (5)	
H11	0.634211	0.790845	0.686230	0.070*	
C14	0.46538 (14)	0.3363 (4)	0.62163 (14)	0.0635 (6)	
H14	0.422987	0.219895	0.604920	0.076*	
C15	0.70221 (14)	0.2747 (4)	0.85021 (13)	0.0671 (6)	
H15A	0.700893	0.127967	0.821055	0.081*	
H15B	0.658921	0.266962	0.871337	0.081*	
C13	0.54145 (14)	0.3061 (4)	0.69666 (13)	0.0619 (5)	
H13	0.549857	0.172823	0.730695	0.074*	
C5	0.13532 (15)	1.1122 (4)	0.19285 (14)	0.0718 (6)	
H5	0.167215	1.243665	0.192412	0.086*	
C16	0.79051 (15)	0.3130 (5)	0.92404 (15)	0.0773 (7)	
H16A	0.832723	0.320157	0.901537	0.093*	
H16B	0.805157	0.179131	0.962854	0.093*	
C2	0.03805 (15)	0.7197 (5)	0.19350 (15)	0.0742 (6)	
H2	0.005409	0.589611	0.193574	0.089*	
C3	0.00415 (16)	0.8855 (5)	0.12943 (16)	0.0789 (7)	
H3	−0.052292	0.867632	0.085531	0.095*	
C4	0.05246 (17)	1.0798 (5)	0.12880 (15)	0.0777 (7)	
H4	0.028216	1.189367	0.084265	0.093*	
C17	0.7977 (2)	0.5324 (5)	0.97463 (17)	0.0988 (9)	
H17A	0.790480	0.667237	0.938607	0.148*	
H17B	0.853871	0.538215	1.024129	0.148*	
H17C	0.753332	0.532467	0.993616	0.148*	

### Atomic displacement parameters (Å^2^)

**Table:**

	*U*^11^	*U*^22^	*U*^33^	*U*^12^	*U*^13^	*U*^23^
S1	0.0643 (3)	0.0685 (4)	0.0671 (3)	−0.0171 (3)	0.0228 (3)	0.0012 (3)
O1	0.0604 (9)	0.0715 (10)	0.0616 (8)	−0.0100 (7)	0.0210 (7)	0.0101 (8)
N2	0.0560 (10)	0.0623 (12)	0.0584 (10)	−0.0022 (9)	0.0246 (9)	−0.0007 (9)
N1	0.0549 (10)	0.0621 (11)	0.0612 (10)	−0.0054 (9)	0.0236 (9)	0.0008 (9)
C7	0.0539 (12)	0.0579 (13)	0.0551 (11)	−0.0053 (10)	0.0271 (10)	−0.0050 (11)
C9	0.0524 (11)	0.0560 (13)	0.0557 (11)	−0.0021 (10)	0.0274 (10)	−0.0045 (10)
C12	0.0536 (11)	0.0575 (13)	0.0550 (11)	−0.0029 (10)	0.0275 (10)	−0.0014 (10)
C8	0.0570 (12)	0.0614 (14)	0.0635 (12)	−0.0044 (11)	0.0305 (11)	−0.0050 (12)
C6	0.0565 (12)	0.0599 (13)	0.0550 (11)	0.0021 (11)	0.0246 (10)	−0.0044 (11)
C1	0.0572 (12)	0.0614 (13)	0.0594 (12)	−0.0056 (10)	0.0270 (10)	−0.0093 (10)
C10	0.0651 (13)	0.0516 (12)	0.0592 (12)	−0.0013 (10)	0.0325 (11)	0.0027 (10)
C11	0.0584 (12)	0.0552 (12)	0.0616 (12)	−0.0137 (10)	0.0282 (11)	−0.0034 (11)
C14	0.0579 (13)	0.0548 (13)	0.0759 (14)	−0.0098 (10)	0.0286 (12)	0.0004 (12)
C15	0.0700 (15)	0.0647 (14)	0.0652 (13)	0.0009 (12)	0.0294 (12)	0.0079 (12)
C13	0.0603 (13)	0.0539 (12)	0.0696 (13)	−0.0032 (11)	0.0278 (11)	0.0088 (11)
C5	0.0758 (16)	0.0652 (15)	0.0683 (14)	−0.0010 (12)	0.0273 (13)	0.0016 (12)
C16	0.0734 (16)	0.0823 (17)	0.0662 (14)	0.0032 (13)	0.0230 (12)	0.0061 (13)
C2	0.0589 (14)	0.0809 (17)	0.0744 (15)	−0.0115 (13)	0.0229 (12)	−0.0076 (14)
C3	0.0581 (13)	0.096 (2)	0.0686 (15)	0.0044 (14)	0.0160 (12)	−0.0107 (15)
C4	0.0792 (16)	0.0786 (17)	0.0643 (13)	0.0142 (15)	0.0229 (13)	0.0020 (13)
C17	0.117 (2)	0.093 (2)	0.0732 (15)	−0.0224 (17)	0.0309 (16)	−0.0068 (15)

### Geometric parameters (Å, º)

**Table:**

S1—C1	1.731 (2)	C14—C13	1.376 (3)
S1—C7	1.770 (2)	C14—H14	0.9300
O1—C12	1.358 (2)	C15—C16	1.495 (3)
O1—C15	1.428 (2)	C15—H15A	0.9700
N2—C8	1.272 (3)	C15—H15B	0.9700
N2—C7	1.378 (2)	C13—H13	0.9300
N1—C7	1.288 (3)	C5—C4	1.367 (3)
N1—C6	1.379 (3)	C5—H5	0.9300
C9—C14	1.384 (3)	C16—C17	1.496 (3)
C9—C10	1.397 (3)	C16—H16A	0.9700
C9—C8	1.444 (3)	C16—H16B	0.9700
C12—C11	1.383 (3)	C2—C3	1.368 (3)
C12—C13	1.385 (3)	C2—H2	0.9300
C8—H8	0.9300	C3—C4	1.387 (4)
C6—C5	1.390 (3)	C3—H3	0.9300
C6—C1	1.395 (3)	C4—H4	0.9300
C1—C2	1.387 (3)	C17—H17A	0.9600
C10—C11	1.364 (3)	C17—H17B	0.9600
C10—H10	0.9300	C17—H17C	0.9600
C11—H11	0.9300		
			
C1—S1—C7	88.68 (10)	O1—C15—H15A	110.1
C12—O1—C15	118.74 (16)	C16—C15—H15A	110.1
C8—N2—C7	120.89 (19)	O1—C15—H15B	110.1
C7—N1—C6	111.07 (18)	C16—C15—H15B	110.1
N1—C7—N2	121.13 (18)	H15A—C15—H15B	108.5
N1—C7—S1	115.23 (15)	C14—C13—C12	119.0 (2)
N2—C7—S1	123.60 (16)	C14—C13—H13	120.5
C14—C9—C10	117.71 (18)	C12—C13—H13	120.5
C14—C9—C8	121.1 (2)	C4—C5—C6	119.1 (2)
C10—C9—C8	121.2 (2)	C4—C5—H5	120.4
O1—C12—C11	115.01 (17)	C6—C5—H5	120.4
O1—C12—C13	125.19 (19)	C15—C16—C17	113.8 (2)
C11—C12—C13	119.79 (19)	C15—C16—H16A	108.8
N2—C8—C9	122.4 (2)	C17—C16—H16A	108.8
N2—C8—H8	118.8	C15—C16—H16B	108.8
C9—C8—H8	118.8	C17—C16—H16B	108.8
N1—C6—C5	124.7 (2)	H16A—C16—H16B	107.7
N1—C6—C1	115.69 (19)	C3—C2—C1	118.3 (2)
C5—C6—C1	119.6 (2)	C3—C2—H2	120.8
C2—C1—C6	121.0 (2)	C1—C2—H2	120.8
C2—C1—S1	129.72 (19)	C2—C3—C4	121.2 (2)
C6—C1—S1	109.32 (15)	C2—C3—H3	119.4
C11—C10—C9	120.7 (2)	C4—C3—H3	119.4
C11—C10—H10	119.6	C5—C4—C3	120.8 (2)
C9—C10—H10	119.6	C5—C4—H4	119.6
C10—C11—C12	120.63 (19)	C3—C4—H4	119.6
C10—C11—H11	119.7	C16—C17—H17A	109.5
C12—C11—H11	119.7	C16—C17—H17B	109.5
C13—C14—C9	122.1 (2)	H17A—C17—H17B	109.5
C13—C14—H14	118.9	C16—C17—H17C	109.5
C9—C14—H14	118.9	H17A—C17—H17C	109.5
O1—C15—C16	107.79 (18)	H17B—C17—H17C	109.5
			
C6—N1—C7—N2	−178.59 (17)	C14—C9—C10—C11	0.6 (3)
C6—N1—C7—S1	−0.9 (2)	C8—C9—C10—C11	−179.48 (19)
C8—N2—C7—N1	−170.83 (19)	C9—C10—C11—C12	−0.8 (3)
C8—N2—C7—S1	11.7 (3)	O1—C12—C11—C10	179.34 (18)
C1—S1—C7—N1	0.44 (17)	C13—C12—C11—C10	0.1 (3)
C1—S1—C7—N2	178.05 (17)	C10—C9—C14—C13	0.5 (3)
C15—O1—C12—C11	176.51 (18)	C8—C9—C14—C13	−179.47 (19)
C15—O1—C12—C13	−4.3 (3)	C12—O1—C15—C16	−177.16 (18)
C7—N2—C8—C9	−178.31 (17)	C9—C14—C13—C12	−1.2 (3)
C14—C9—C8—N2	171.56 (19)	O1—C12—C13—C14	−178.23 (19)
C10—C9—C8—N2	−8.4 (3)	C11—C12—C13—C14	1.0 (3)
C7—N1—C6—C5	−178.5 (2)	N1—C6—C5—C4	179.4 (2)
C7—N1—C6—C1	1.1 (2)	C1—C6—C5—C4	−0.2 (3)
N1—C6—C1—C2	−179.90 (19)	O1—C15—C16—C17	61.6 (3)
C5—C6—C1—C2	−0.3 (3)	C6—C1—C2—C3	0.3 (3)
N1—C6—C1—S1	−0.7 (2)	S1—C1—C2—C3	−178.68 (18)
C5—C6—C1—S1	178.91 (16)	C1—C2—C3—C4	0.1 (4)
C7—S1—C1—C2	179.3 (2)	C6—C5—C4—C3	0.6 (4)
C7—S1—C1—C6	0.16 (15)	C2—C3—C4—C5	−0.6 (4)

### Hydrogen-bond geometry (Å, º)

*Cg*2 is the centroid of the C1–C6 phenyl ring.

**Table:**

*D*—H···*A*	*D*—H	H···*A*	*D*···*A*	*D*—H···*A*
C11—H11···N1^i^	0.93	2.49	3.362 (3)	157
C16—H16*A*···*Cg*2^ii^	0.97	2.91 (2)	3.765 (3)	147

Symmetry codes: (i) −*x*+1, −*y*+2, −*z*+1; (ii) −*x*+1, −*y*+2, −*z*.
